# A Multicenter, Randomized, Controlled, Clinical Trial Evaluating a Lyopreserved Amniotic Membrane in the Treatment of Venous Leg Ulcers

**DOI:** 10.1002/hsr2.70819

**Published:** 2025-05-04

**Authors:** Yadwinder Dhillon, Lena Levine, Gregory Tovmassian, Alexander Reyzelman, Francisco Perez‐Clavijo, Francis Wodie, Shawn Cazzell, Allan Grossman, Lesly Robinson, Felix Sigal, Robert S Kirsner, Mher Vartivarian, Molly Saunders, Jaideep Banerjee

**Affiliations:** ^1^ Titan Clinical Solutions Phoenix Arizona USA; ^2^ Acclaim Bone & Joint Institute Fort Worth Texas USA; ^3^ Center for Clinical Research Carmichael California USA; ^4^ Center for Clinical Research Castro Valley California USA; ^5^ Integral Clinical Trials Solutions Doral Florida USA; ^6^ Integral Clinical Trials Solutions Homestead Florida USA; ^7^ Limb Preservation Platform Fresno California USA; ^8^ Harrisburg Foot and Ankle Center Harrisburg Pennsylvania USA; ^9^ Temple University School of Podiatric Medicine Philadelphia Pennsylvania USA; ^10^ LA Foot Pain and Ankle Clinic Los Angeles California USA; ^11^ University of Miami Miami Florida USA; ^12^ Center for Clinical Research San Francisco California USA; ^13^ Osiris Therapeutics Columbia Maryland USA; ^14^ Smith & Nephew Inc. Fortworth Texas USA

**Keywords:** allografts, amniotic membrane, quality of life, skin substitute, venous ulcers

## Abstract

**Background:**

Standard of Care (SoC) with multilayer compression therapy along with proper wound management, may not be sufficient to close all venous ulcers and needs advanced therapies.

**Methods:**

In this multicenter, prospective, randomized, controlled, open‐label trial, 351 patients were screened, 200 were eligible and enrolled and were randomized 1:1 to LPM (lyopreserved cellular placental membrane) plus SoC or SoC alone for up to 12 weeks. Patients were enrolled between June 2018 and November 2020 at 30 sites across the United States. Outcome measures included complete closure of the index ulcer (primary), reduction in wound size, rate of closure, quality of life, and adverse events.

**Results:**

ITT analysis revealed that wounds treated with weekly applications of LPM as an adjunct to standard of care, reduced in size significantly more than SoC alone, at the end of 4, 8, and 12 graft applications, indicating a faster progression to closure. There was a statistically 1.72 higher relative risk or 72% higher probability of wound closure with LPM compared to the SoC group during the study period for wounds with an initial size of 3–25 cm^2^. Use of LPM as an adjunct was able to close statistically larger‐sized wounds on average. There was also a statistically significant fivefold improvement in quality of life (overall physical symptoms and daily life) over baseline, in the LPM treated patients as compared to the control group.

**Conclusion:**

LPM and standard of care, significantly closed more venous leg ulcers and faster than standard of care alone and improved the quality of life for patients, suggesting that the use of aseptically processed LPM is a safe and effective treatment option in the healing of chronic venous leg ulcers.

**Trial Registration:** ClinicalTrials.gov ID: NCT03629236, Study to Evaluate Safety and Efficacy of GrafixPL for the Treatment of Venous Leg Ulcers. (https://clinicaltrials.gov/study/NCT03629236).

**Abbreviations**
ANCOVA: analysis of covariance
CAMP: cellular and acellular matrix‐based products
CPM: cryopreserved placental membrane
ITT: intent‐to‐treat
LPM: lyopreserved placental membrane
QoL: quality of life
SoC: standard of care
TEAE: treatment associated adverse events
VLU: venous leg ulcer

## 1 Introduction

Venous leg ulcers (VLUs) are a type of chronic wound resulting from venous insufficiency. A mismatch of venous pressure and unidirectional blood flow contributes to the underlying pathophysiology of venous ulcers [1]. Microcirculatory dysfunction due to dilated capillaries and reduced capillary density, valvular incompetence or faulty veins and a resultant venous hypertension has been shown to be negatively associated with VLUs and leads to diminished tissue oxygenation and breakdown [2, 3]. A perpetuated chronic skin inflammation and insufficient granulation are often a characteristic of venous ulcers [4], making them some of the hardest wounds to heal.

The standard approach to treatment of VLUs includes debridement, wound dressings, and aggressive compression therapy [5, 6]. Compression therapy aids in healing by improving venous return and skin blood flow and reduces edema by opposing leakage of fluid from capillaries into tissue [5, 6]. With good wound care and compression therapy, VLUs usually heal within 6 months [7]. However, larger wound size and longer ulcer durations, among other factors, have been consistently identified as major factors associated with delayed healing of VLUs with standard therapy alone [7, 8].

Incorporation of advanced therapies such as cellular and acellular matrix‐based products (CAMPs) into the treatment plan have been shown to facilitate wound closure in chronic ulcers [9, 10, 11]. One such commercially available product in the USA, is a cellular amniotic membrane allograft which can be either cryopreserved (CPM, cryopreserved placental membrane), or shelf stable (LPM, lyopreserved placental membrane), which can be used as a wrap, cover or barrier on wounds of different etiologies including DFUs and VLUs. Both CPM and LPM retain native growth factors and cells of the placenta [12]. CPM and LPM has been reported to be clinically equivalent [12, 13, 14, 15].

Positive clinical outcomes for CPM and LPM in the treatment of wounds of various etiologies, including DFUs and VLUs have been reported in multiple prospective and retrospective clinical studies. In a prospective study [16], refractory VLU patients received applications of CPM adjunct to standard care. Complete ulcer healing was achieved in 53% of the VLUs with a mean reduction in wound surface area by 79% in an average of 10.9 weeks. In another prospective study, VLU wounds were reported to close in an average of 42 days [17]. Other retrospective studies have also reported the effectiveness of CPM or LPM in the closure of VLU wounds [18, 19]. In a comparative effectiveness study [20], 70% of wounds were reported to close with the application of CPM as compared to 7.1% with the application of a dehydrated amnion‐chorion membrane. Finally, in a retrospective cohort study [11], using real‐world evidence from 122,012 VLU episodes among 80,415 beneficiaries in the U.S. Medicare population between 2016 and 2020, CPM and LPM treated patients were associated with reduced 1‐year mortality (−23%), reduced recurrence (−80%), and 66.77% reduction in adverse outcomes as compared to standard care. These allografts were also associated with a 73% reduced risk of recurrence of VLUs, compared to other commonly used CAMPs.

This randomized controlled trial is the first Level 1 evidence for LPM and evaluates the safety and efficacy of LPM on wound closure and quality of life in chronic VLUs.

## 2 Materials and Methods

LPM (GrafixPL PRIME, Smith & Nephew, Columbia, MD) is a shelf‐stable lyopreserved amniotic membrane allograft that retains the extracellular matrix (ECM), growth factors and endogenous cells of the native placental tissue. LPM is aseptically processed from donated human placental tissue, stored at room temperature and distributed for use in accordance with the regulations outlined in 21 Code of Federal Regulations (CFR) 1271 and the standard of the American Association of Tissue Banks (AATB). LPM has a shelf life of 2.5 years.

### 2.1 Study Design and Patient Population

The present study is a multi‐center, prospective, randomized, open‐label trial to evaluate the efficacy of LPM plus standard compression therapy for the treatment of chronic VLUs (ClinicalTrials.gov ID: NCT03629236). Patients were enrolled between June 2018 and November 2020 at 30 sites across the United States.

Standard ulcer care: Ulcers were appropriately cleaned and debrided. Surgical or sharp debridement were employed to remove all nonviable soft tissue from the ulcer by scalpel, tissue nippers and/or curettes, per investigator discretion. Autolytic and enzymatic debridement techniques were not allowed. All patients had their index ulcer wrapped with multi‐layer compression dressing by the treating investigator. An ulcer area measurement was manually calculated using a ruler at the Baseline Visit, in addition to the ulcer area measurement automatically calculated by the eKare photograph system (eKare inSight 3D wound imaging system and acetate tracing).

LPM is a commercially available product (GRAFIX PL PRIME, Smith & Nephew Inc., Columbia, MD, USA). Two hundred patients (100 in each treatment group) were randomized at a 1:1 ratio to LPM plus standard compression therapy or standard compression therapy alone. Patients randomized to the treatment group received applications of LPM plus standard compression therapy once weekly for up to 12 weeks. Both group of patients returned for weekly visits until the ulcer was closed or until the End of Treatment Visit. A closed ulcer was defined as 100% re‐epithelialization without drainage or dressing. Outcome measures included complete closure of the index ulcer (primary), time to initial ulcer closure, percentage area reduction in ulcers that did not achieve closure and number and type of AEs and SAEs. A crossover extension phase of the study will be analyzed and reported in a follow‐up manuscript.

### 2.2 Sample Size Calculation and Treatment Assignments

It was anticipated that approximately 45% of subjects in the LPM plus standard compression therapy group and 25% of subjects in the standard compression therapy alone group will experience complete ulcer closure at the end of the study, based on reported evidence from RCTs with CAMPs in the US VLU population at the time of the study design (15%–35% for SoC arm and 38%–60% for treatment arm indicating an effect size of 15%–25%) [21, 22, 23, 24, 25]. This sample size was thought to be sufficient to provide 80% power for the two‐sided significance level of 0.05 based on the *χ* ^2^ test with continuity correction (nQuery Advisor). Based on this, approximately 200 patients (100 in each group) were planned to be enrolled in the trial. It was expected that 196 will be eligible for the evaluable population. Patients were initially randomized (1:1) to either LPM plus standard compression therapy or standard compression therapy alone in accordance with a central randomization schedule using Interactive Web Response System (IWRS), generated by CPC Clinical Research and implemented by DATATRAK.

### 2.3 Study Population

Patients were enrolled from 30 centers located in the US (See “The Grafix Venous Leg Ulcer Study Group” in Section Acknowledgments). The study population consisted of male and female patients, 18 years or older, who had a lower extremity chronic VLU. The Index Ulcer, identified by the Investigator, was defined as an open ulcer area between 1 and 25 cm^2^ inclusive that has been present for at least 4 weeks at the time of screening.

### 2.4 Inclusion and Exclusion Criteria

The inclusion and exclusion criteria are listed in Table 1.

**Table 1:** Inclusion and exclusion criteria.

Inclusion criteria	Exclusion criteria
1. 18 years or older, as of the date of screening. 2. An Index Ulcer that is chronic (defined as present for > 4 weeks, but not present for more than 52 weeks at Screening Visit 1). 3. Index Ulcer is located on the leg, below the knee and above the malleoli (ulcer may be inclusive of the malleoli). 4. The Index Ulcer is between 1 and 25 cm^2^, inclusive, at the Screening and Baseline Visits. The longest dimension of the index ulcer cannot exceed 10 cm at the Baseline Visit. 5. The Index Ulcer has had compression therapy for > 2 weeks at Screening Visit 1. 6. The Index Ulcer extends into the dermis or subcutaneous tissue without evidence of exposed muscle, tendon, bone, or joint capsule. 7. Patient has adequate circulation to the foot, as documented up to 14 days before Screening Visit 1 or during the screening period by either: o Ankle Brachial Index (ABI) > 0.80 and < 1.30, or o In patients with noncompressible ankle vessels defined as an ABI ≥ 1.30, a Toe Brachial Index (TBI) ≥ 0.50, or o In patients with noncompressible ankle vessels defined as an ABI ≥ 1.30 and TBI cannot be performed (e.g., toe is absent, ulcers are present, or site cannot perform a TBI), a Doppler waveform in the posterior tibial or dorsalis pedis arteries at the ankle consistent with adequate flow in the foot (biphasic or triphasic) or other diagnostic confirmation of adequate flow (e.g., duplex imaging, normal pulse volume recording [PVR] testing). 8. Confirmed venous insufficiency, as documented up to 30 days before enrollment (Baseline Day 0), by either: o Duplex ultrasonography, or o Principal Investigator [PI] clinical assessment to include clinical signs and symptoms of venous ulcerations (e.g., hyperpigmentation of surrounding skin, varicosities, and/or lipodermatosclerosis).	1. Index Ulcer is of non‐venous pathophysiology. 2. Gangrene is present on any part of the affected limb. 3. Patient is unable to tolerate standard compression therapy. 4. Glycated hemoglobin A1c (HbA1c) level of > 14% in any patient with type 1 or type 2 diabetes mellitus, as documented up to 14 days before Screening Visit 1 or during the screening period. 5. Patient is receiving corticosteroids, immunosuppressive or cytotoxic agents intravenously (IV) at any time during the screening period. 6. Patient has an ulcer due to any cause within 5 cm of the Index Ulcer identified for study consideration at Baseline. 7. Patient is Human Immunodeficiency Virus (HIV) positive or has Acquired Immune Deficiency Syndrome (AIDS). 8. Current evidence of infection at the Index Ulcer, including cellulitis and/or pus drainage from the ulcer site at the time of Screening and Baseline Visits. 9. Evidence of osteomyelitis at the time of Screening and Baseline Visits. 10. Patient has active malignancy other than non‐melanoma skin cancer. 11. Patient's Index Ulcer has decreased by ≥ 30% between Screening Visit 1 and the Baseline Visit during the screening period. 12. Patient has untreated alcohol or substance abuse at the time of Screening Visit 1. 13. Pregnant women and women who are breastfeeding. 14. Patient is currently enrolled in or has participated in another investigational device, drug, or biological trial within 30 days before Screening Visit 1. 15. Patient has had, within 14 days of Screening Visit 1, or is currently undergoing, or is planning for ulcer treatments with growth factors, living skin, dermal substitutes or other advanced biological therapies. 16. Patient is an employee, or an immediate family member of an employee, of the sponsor company or site research staff conducting the study. 17. Patients with a history of poor compliance, or an unwillingness or inability to adhere to the requirements of the protocol.

### 2.5 Patient Screening

All patients who signed an institutional review board (IRB) approved informed consent form (ICF) and an authorization for use of protected health information (PHI) were assigned a unique screening number consisting of a two‐digit site number followed by a three‐digit patient number. If a patient failed screening and did not receive treatment, s/he could be re‐screened once at the discretion of the Investigator, after waiting at least 14 days from the beginning of the first screening period. Patients were assigned a new study identification number on re‐screening. Patients could not be re‐screened more than one time within any consecutive 12‐month period.

### 2.6 Study Visits

Table 2 describes the study visit schedule. LPM plus standard compression therapy was administered once a week (± 3 days) starting with the Baseline Visit for 12 weeks during the Treatment Phase. The 5 × 5 cm size for the graft was used and the ulcer was covered in its entirety with product, up to 25 cm^2^. No other ulcer care products were given during the study. Excluded concomitant treatments include HBO, NPWT, enzyme therapy for debridement, growth factors, dermal substitutes, or other advanced biologic therapies. Patients with an infected index ulcer could continue in the study if the infection could be managed with oral or IV antibiotics.

**Table 2:** Schedule of study visits.

	Screening visit 1 Day 14	Screening visit 2 Day 7	Baseline and initial treatment visit Day 0	Treatment visits ^a^, ^b^ up to Day 77 after baseline visit	End of treatment visit^c^ up to Day 84 after baseline visit	Follow‐up visits^d^ Days 7, 14, 21, 28, 84, after end of treatment visit	Early termination visit
Visit window		7 Days from Screening visit 1 (+ 3 days)	7 Days from Screening visit 2 (+ 3 days)	Weekly from Baseline visit (± 3 days)	7 Days from Treatment Visit (± 3 days)	Weekly for month 1 (± 3 days); months 3 and 6 (± 7 days) from end of treatment visit	Anytime patient exits trial
Total number of visits	1	1	1	Up to 11	1	Up to 6	1
Informed consent/PHI authorization	×						
Demographics	×						
Medical history	×	×	×				
Concomitant medications and procedures assessment	×	×	×	×	×	×	×
Physical exam, height and weight	×						
Vital signs	×	×	×	×	×	×	×
HbA1c, urine pregnancy	×^e^						
ABI, TBI or Doppler waveform	×						
Standard ulcer care and compression therapy	×	×	×	×		×^j^	
Ulcer swab sample collection			×	×^f^			
CWIS and work productivity and activity impairment (WPAI) questionnaire			× (before treatment)	× (visits 5 and 9 only)	×	× (visits 4,5, and 6 only)	×
eKARE ulcer photograph including ulcer area measurement	×	×	×	×	×	×	×
Acetate tracing	×	×	×	×	×		×
Manual ulcer area measurement			×^g^				
Determination of % ulcer improvement		×^h^	×^h^				
Inclusion/exclusion determination	×	×^i^	×^i^				
Randomization			×				
Ulcer treatment			×	×			
Adverse events assessment			×^k^	×	×	×	×

^a^ If a patient needed to be seen for treatment between weekly visits due to disruption of the dressing, etc. an unscheduled visit was conducted at the discretion of the investigator.

^b^ If the ulcer is closed before 12 visits, patients will instead complete the End of Treatment Visit.

^c^ Completed by patients whose index ulcer has (1) achieved complete ulcer closure (defined as 100% re‐epithelialization of the index ulcer as determined by the Investigator) or (2) did not achieve closure after completion of Baseline and all Treatment Visits.

^d^ Completed by patients who achieve complete ulcer closure and complete the End of Treatment Visit. Patients will be provided a compression stocking at the End of Treatment Visit, to be worn at all times while at home.

^e^ HbA1c blood draws only in patients with type 1 or type 2 diabetes.

^f^ Ulcer swab samples collected only at Treatment Visit 2 (Day 7) and Treatment Visit 4 (Day 21) at designated sites.

^g^ An ulcer area measurement was manually calculated using a ruler at the Baseline Visit, in addition to the ulcer area measurement automatically calculated by the eKare photograph system.

^h^ If ulcer area has decreased by ≥ 30% from Screening Visit 1, the patient did not meet study eligibility criteria.

^i^ Exclusion Criteria #11 was only evaluated at Screening Visit 2 and Baseline Visit. All other criteria were evaluated at both Screening Visits and Baseline Visit.

^j^ Compression dressings removed, ulcer assessed and confirmed for closure, and patient provided with a compression stocking to be worn every day up to Follow‐Up Visit.

^k^ At time of and after time of ulcer treatment.

### 2.7 Definition of Adverse Event and Serious Adverse Event

An AE was defined as any untoward medical occurrence in a patient, which does not necessarily have to have a causal relationship with this treatment. The severity of an AE was determined by the Investigator or reported by the patient. An SAE includes any AE that results in any of the following outcomes: (1) death; (2) life‐threatening; (3) persistent or significant incapacity or substantial disruption of the ability to conduct normal life functions; (4) required inpatient hospitalization or prolonged hospitalization; (5) congenital anomaly or birth defect; (6) any medically significant events that may require medical or surgical intervention to prevent one of the outcomes listed above.

### 2.8 Exploratory Endpoints: Quality of Life

Change in overall well‐being and work Productivity from Baseline, was determined by the Cardiff Wound Impact Schedule (CWIS) [26].

### 2.9 Statistical Analysis

ITT population was used to analyze the data. Statistical tests were conducted against a two‐sided alternative hypothesis, employing a significance level of 0.05. Risk ratio analysis was performed by using complete wound closure as the risk and treatment with LPM and control (without LPM) as the variable. Risk ratio was calculated as RR = (a/(a + b))/(c/(c + d)), where: *a* = number of closed wounds in the LPM group, *b* = number of unhealed wounds in the LPM group, *c* = number of closed wounds in the control group, *d* = number of unhealed wounds in the control group. Subgroup analysis was performed with initial wound size as the variable. This was based on literature that initial wound size can affect VLU wound closure rates [27, 28]. Stratified relative risk analysis was done by calculating Mantel–Haenszel pooled relative risk and Cochran–Mantel–Haenszel (CMH) test was used to test whether there's a consistent independent or association between two variables (treatment and outcome) across multiple strata (wound size). The null hypothesis of the CMH test was that there is no association between the two variables (treatment and outcome) across the strata, after adjusting for the stratifying variable. To further have confidence on the interpretation from the subgroup analysis, a sensitivity analysis was performed to see if the risk of wound closure remained statistically significant, even after eliminating the wounds of smaller initial wound size. Kaplan–Meier's (K–M) survival curve was also used to compare wound closure outcomes between the study groups and the log rank test was used to test if there is significant difference in survival distributions. Mann–Whitney U test and Mood's Median test was used to compare rates and incidences between the study groups. SRplot was used to generate the forest plot. SAS 9.4 (SAS institute Inc., Cary, NC) or Microsoft Excel Data Analysis ToolPak were used for analysis. Change in wound size in a week was used to identify outliers and trimming was applied to exclude samples beyond the 99.9 percentile in both arms.

### 2.10 Ethics and Responsibility

All participants gave informed consent for participation in the study. The study was approved by the IRB of each treating institution. All aspects of the study will be conducted in accordance with GCP as described in the ICH Guideline (E6) and CFR ICH Selected Regulations and Guidance for Drug Studies, Sections 50, 54 and 56 of CFR Title 21 and all applicable national and local regulations at each study site. Patient monitoring was conducted according to GCP and standard operating procedures in compliance with applicable government regulations. Individual study sites were be monitored at appropriate intervals based on a detailed clinical monitoring plan to ensure satisfactory enrollment, data recording, and adherence to the protocol. The study was registered with ClinicalTrials.gov (https://clinicaltrials.gov/study/NCT03629236).

## 3 Results

**Figure 1:**
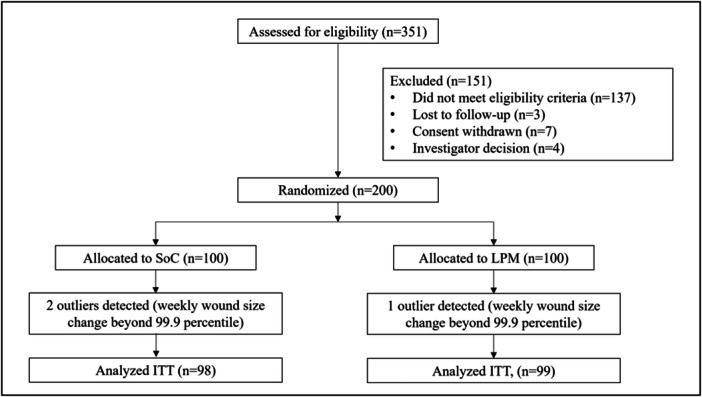
CONSORT flowchart.

A total of 351 subjects were screened and entered the study for the 2‐week run‐in period between November 2018 and June 2020. At the end of the screening phase, 151 patients were no longer eligible for randomization. One hundred patients were randomized to the LPM group and 100 to the standard care group. (Figure 1).

### 3.1 Patients and Wounds Baseline Characteristics

The patients' baseline characteristics and initial wound size **(**Table 3 **)** were similar in both group of patients at enrollment. Age of the patients ranged from 22 to 90 years old with a median age of 62.5 in the control group and 63.5 in the LPM group. The demographics of the patient population recruited in this study also align with the race and ethnicity of the US national Medicaid and CHIP population [29]. The mean wound size was 5.87 in the control group and 5.97 in the LPM group.

**Table 3:** Baseline wound characteristics.

Demographics		Control	LPM	*p‐*value
Age at informed consent (years)	Mean (SD)	62.3 (13.49)	63.2 (12.15)	ns
	Median (min, max)	62.5 (22, 90)	63.5 (23, 88)	
Age group (%)	≤ 18 years	0	0	ns
	19–64 years	56%	52%	
	≥ 65 years	44%	48%	
Sex (%)	Male	61%	62%	ns
	Female	39%	38%	
Ethnicity (%)	Hispanic or Latino	27%	31%	ns
	Not Hispanic or Latino	72%	67%	
	Not Reported	0	1%	
	Unknown	1%	1%	
Race (%)	Black or African American	22%	27%	ns
	American Indian or Alaskan Native	2%	0	
	Asian	0	1%	
	Native Hawaiian or Other Pacific Islander	2%	0	
	White	74%	67%	
	Not reported	0	3%	
	Unknown	0	2%	
Initial wound size (cm^2^)	Mean (SD)	5.87 (5.2)	5.95 (4.56)	ns
	Median (min, max)	3.6 (1, 22.8)	4.6 (0.8, 20.6)	

### 3.2 Incidence of Complete Wound Closure and Follow‐Up

**Figure 2:**
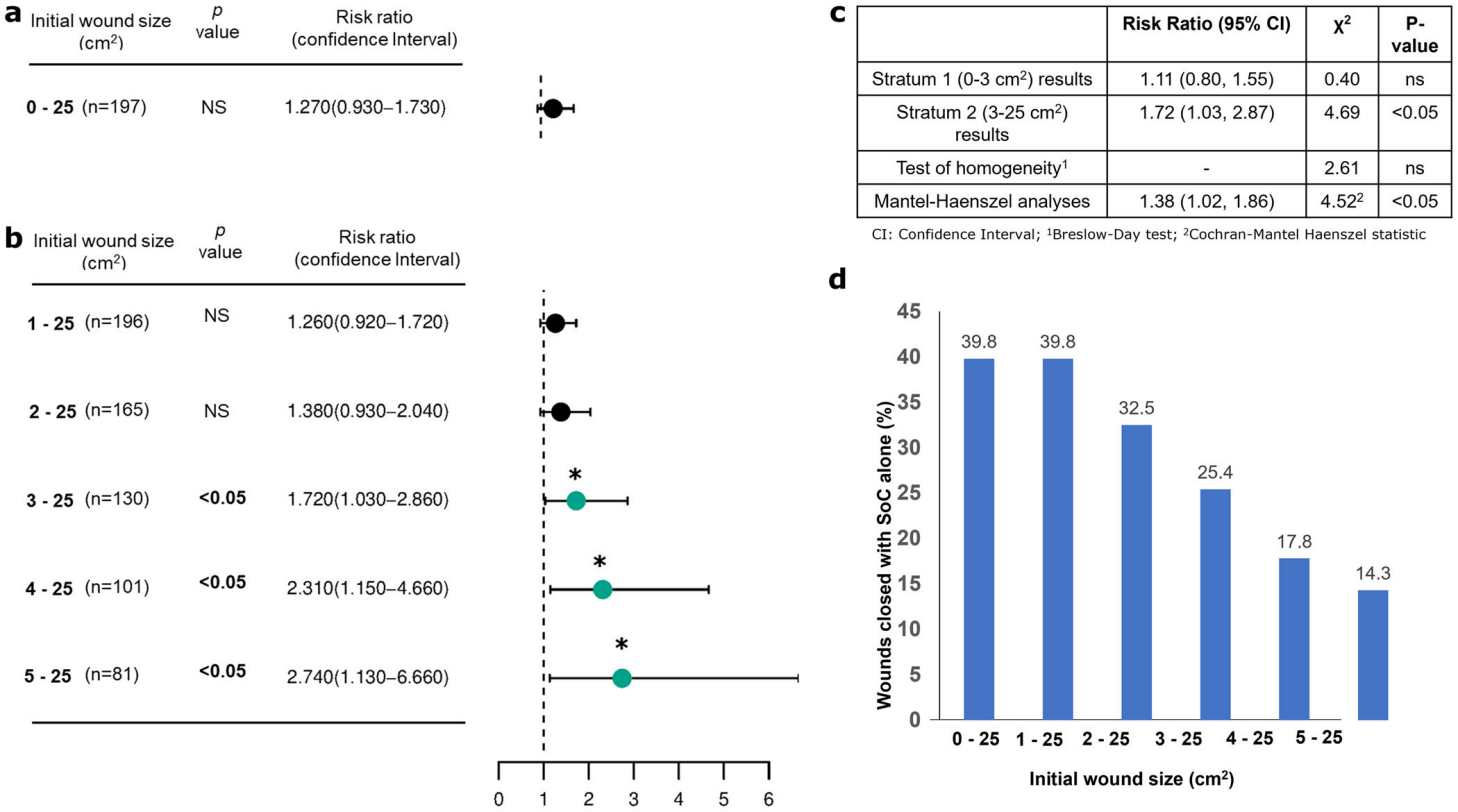
Quantitative analysis of wound closure. (a) Probability (risk ratio) of complete wound closure for all wounds in LPM over control group. (b) Subgroup analysis using initial wound size as variable. (c) Stratified risk ratio analysis. (d) Decrease in efficacy of SoC with increasing initial wound size; (*ns*=not significant). *p < 0.05.

Results from the ITT population show that there was 1.27 higher relative risk or 27% more probability of wound closure with LPM compared to the control group, however, this was not statistically significant (Figure 2a). Subgroup analysis demonstrated that there was a statistically 1.72 higher relative risk or 72% more probability of wound closure (95% CI, 1.03–2.86, *p* < 0.05) with LPM compared to the control group during the study period for wounds with an initial size 3–25 cm^2^. Sensitivity analysis demonstrated that the higher risk of wound closure remained statistically significant, even after eliminating the wounds of smaller initial wound size, the risk ratio being 2.31 for wounds 4–25 cm^2^ (95% CI, 1.15–4.66, *p* < 0.05) and 2.74 for wounds 5–25 cm^2^ (95% CI, 1.13–6.66, *p* < 0.05) (Figure 2b). However, for wounds between 0 and 3 cm^2^, the risk of outcome (closure) in the LPM group was 1.11 times the risk in the control group and the association was not statistically significant (Figure 2b). Hence, when wounds < 3 cm^2^ were included in the analysis, LPM treated wounds failed to reach a statistically significant improvement. Stratified risk ratio analysis provided further confirmation of the results. The *p *< 0.05 of the Cochran–Mantel–Haenszel (CMH) test suggested that there was an association between LPM and outcome (wound closure) in at least one stratum. Additionally, the CMH‐adjusted risk ratio was significant (risk ratio: 1.38; 95% CI: 1.02–1.86) after adjusting for stratification. Calculation of the pooled or Mantel‐Haenszel risk ratio was justified as the results of the Breslow‐Day test suggested that there was not enough evidence to detect a difference in association between the two strata (Figure 2c).

We noted that with SoC alone, wound closure rate decreases with increase in initial wound size (Figure 2d). Therefore, the data reveals that, as the efficacy of SoC to close wounds, decreases with increasing wound size (Figure 2d), addition of LPM improves the efficacy and becomes statistically significant beyond an initial wound size of 3 cm^2^.

**Figure 3:**
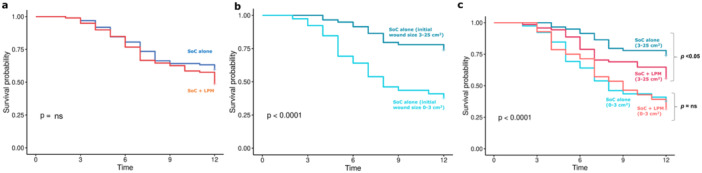
Kaplan–Meier analysis for probability of closure with (a) SOC alone vs. SOC with LPM for all wounds, (b) SOC alone stratified for initial wound size of 0–3 cm^2^ and 3–25 cm^2^, (c) SOC alone vs. SOC with LPM, stratified for initial wound size of 0–3 cm^2^ and 3‐25 cm^2^; (*ns* = not significant).

K–M curve demonstrates wound closure distribution for all wounds over 12 weeks (39.8% for control vs. 50.5% for LPM group) (Figure 3a). For wounds with an initial size of 0–3 cm^2^, SoC alone was able to close 61.5% of the wounds, while for wounds 3–25 cm^2^, SoC alone was able to close only 25.4% of the wounds at 12 weeks (Figure 3b). Addition of LPM was able to improve the wound closure rate to 67.9% and 43.6% (*p* < 0.05), respectively. While the medical necessity for the adjunct treatment with LPM in the smaller wounds, which are open for less than a year, is thus not evident, addition of LPM as an adjunct therapy, statistically closes a greater number of wounds and becomes clinically beneficial beyond a certain initial wound size and as SoC becomes progressively less effective (Figure 3c).

For all the wounds that healed, recurrence recorded at 3 months post‐closure occurred for 9 out of 49 wounds (18.4%) for the LPM group and 9 out of 40 wounds (22.5%) for the control group.

### 3.3 Reduction in Wound Area and Rate of Closure

**Figure 4:**
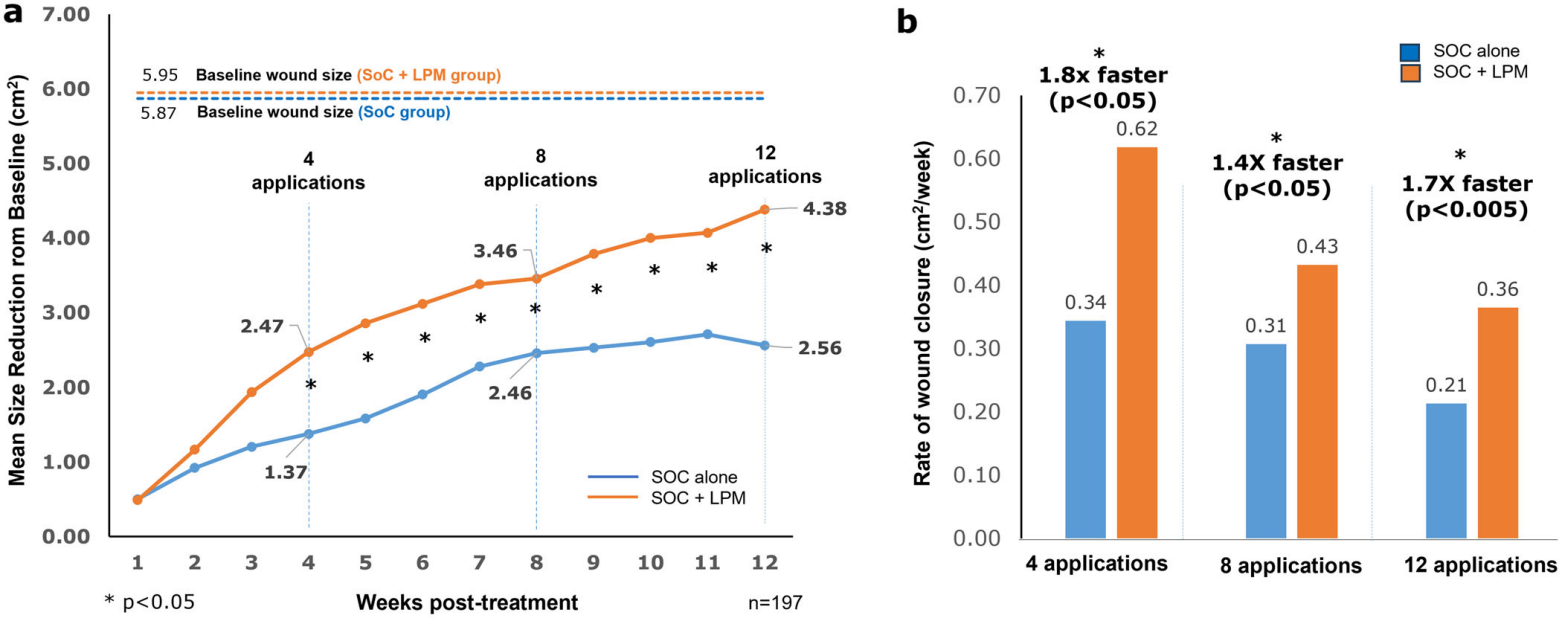
Progression of wound healing. (a) Mean absolute reduction in wound size from baseline. (b) Rate of wound closure after 4, 8, and 12 applications. *p < 0.05.

**Figure 5:**
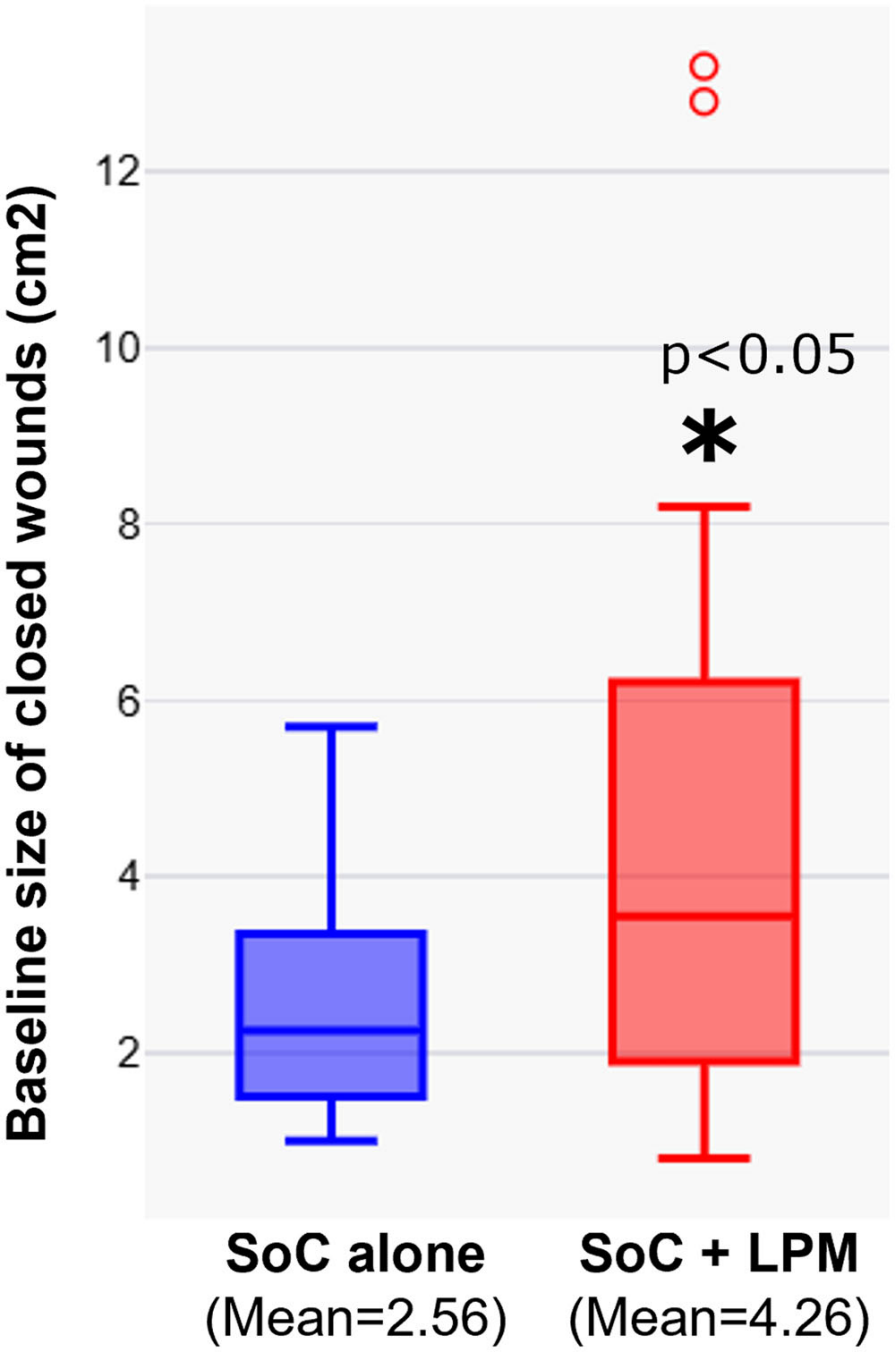
Distribution of initial wound size for closed wounds. *p < 0.05.

Wounds reduced on an average of 1.37 cm^2^ (23.4% of mean initial size) in the control group vs 2.47 cm^2^ (41.5%) in the treatment group (*p* < 0.05). 87.9% of the wounds treated with LPM, showed reduction in wound size or progress towards closure, after 4 applications. After 8 applications, the average reduction was 2.46 cm^2^ (41.9%) for the control group vs. 3.46 cm^2^ (58.1%) for the treatment group (*p* < 0.05). After 12 applications, the average reduction was 2.56 cm^2^ (43.6%) for the control group vs. 4.38 cm^2^ (73.6%) for the treatment group (*p *< 0.005) (Figure 4a). The rates of wound progression towards closure were significantly faster with application of LPM (80%, 40%, and 70% faster after 4, 8, and 12 applications, respectively) (Figure 4b). Figure 5 plots the distribution of wound sizes that are closed by SoC alone and SoC + LPM. LPM was able to close statistically larger sized wounds.

### 3.4 Safety

Total of 31 treatment emergent adverse events (TEAE) in the index ulcer were reported. Seventeen were in the control group and 14 in the LPM group (Table 4). Ten treatment emergent serious adverse events were reported. Five were in the control group and 5 in the LPM group **(**Table 5 **)**.

**Table 4:** Treatment emergent adverse events in the index ulcer.

System organ class preferred term	LPM + SOC (*N* = 100)	SOC alone (*N* = 100)
Number (%) of subjects with at least one TEAE in the index ulcer	14 (14.0%)	17 (17.0%)
Infections and infestations	6 (6.0%)	8 (8.0%)
Cellulitis	2 (2.0%)	5 (5.0%)
Infected skin ulcer	3 (3.0%)	1 (1.0%)
Localized infection	0	1 (1.0%)
Soft tissue infection	0	1 (1.0%)
Wound infection	1 (1.0%)	0
Skin and subcutaneous tissue disorders	8 (8.0%)	5 (5.0%)
Skin ulcer	3 (3.0%)	2 (2.0%)
Dermatitis	0	2 (2.0%)
Blister	1 (1.0%)	0
Folliculitis	1 (1.0%)	0
Pruritus	1 (1.0%)	0
Skin lesion	1 (1.0%)	0
Statis dermatitis	0	1 (1.0%)
Venous ulcer pain	1 (1.0%)	0
Injury, poisoning and procedural complications	1 (1.0%)	3 (3.0%)
Skin abrasion	0	2 (2.0%)
Skin pressure mark	0	1 (1.0%)
Wound complication	1 (1.0%)	0
Musculoskeletal and connective tissue disorders	1 (1.0%)	3 (3.0%)
Pain in extremity	1 (1.0%)	2 (2.0%)
Arthralgia	0	1 (1.0%)
Immune system disorders	0	1 (1.0%)
Reaction to excipient	0	1 (1.0%)

**Table 5:** Treatment emergent serious adverse events.

System organ class preferred term	LPM + SOC (*N* = 100)	SOC alone (*N* = 100)
Number (%) of subjects with at least one TESAE	5 (5.0%)	5 (5.0%)
Infections and infestations	2 (2.0%)	2 (2.0%)
Abscess limb	0	1 (1.0%)
Bronchitis	1 (1.0%)	0
Corona virus infection	0	1 (1.0%)
Upper respiratory tract infection	1 (1.0%)	0
Cardiac disorders	2 (2.0%)	1 (1.0%)
Acute myocardial infarction	0	1 (1.0%)
Bradycardia	1 (1.0%)	0
Myocardial infarction	1 (1.0%)	0
Injury, poisoning and procedural complications	0	1 (1.0%)
Fall	0	1 (1.0%)
Investigations	0	1 (1.0%)
Laboratory test abnormal	0	1 (1.0%)
Metabolism and nutrition disorders	1 (1.0%)	0
Electrolyte imbalance	1 (1.0%)	0
Musculoskeletal and connective tissue disorders	0	1 (1.0%)
Rhabdomyolysis	0	1 (1.0%)
Respiratory, thoracic and mediastinal disorders	0	1 (1.0%)
Chronic obstructive pulmonary disease	0	1 (1.0%)

### 3.5 Quality of Life

**Figure 6:**
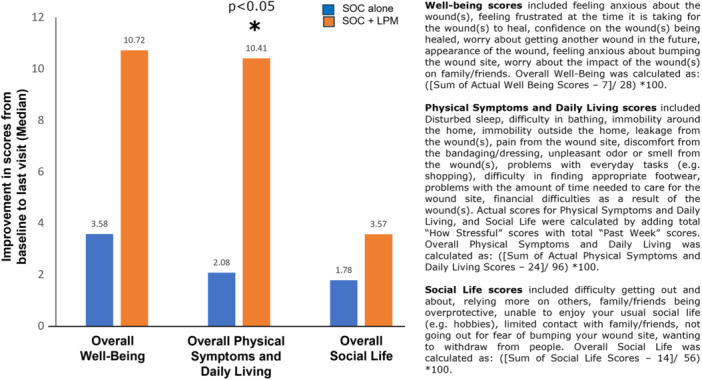
Improvement in quality of life (QoL) scores from baseline to last visit. *p < 0.05.

Improvement in overall physical symptoms and daily life over baseline, was significantly more in the LPM treated patients as compared to the control group (fivefold, *p* < 0.05). Physical Symptoms and Daily Living scores included disturbed sleep, difficulty in bathing, immobility around the home, immobility outside the home, leakage from the wound(s), pain from the wound site, discomfort from the bandaging/dressing, unpleasant odor or smell from the wound(s), problems with everyday tasks (e.g., shopping), difficulty in finding appropriate footwear, problems with the amount of time needed to care for the wound site and financial difficulties as a result of the wound(s). Overall well‐being and overall social life trended towards improvement in the LPM group but was not statistically significant (Figure 6).

## 4 Discussion

Multilayer compression is considered SoC for VLUs [30]. However, it is also well known that SoC alone is not always effective and 40%–70% of the wounds remain unhealed after 6 months and 15%–30% of wounds after a year of care [31]. Limited success has been achieved with conventional wound dressings, such as hydrocolloids, gauze, foams, and films, for the treatment of VLUs and alternative approaches are needed. The nonoperative approach includes lifestyle modification, exercise, compression, and pharmacologic therapies. The Addition of topical hyaluronic acid to a protocol of care that included standardized elastic compression has been shown to improve healing outcomes of VLUs [32, 33]. Exercise has been shown to be beneficial in patients with venous leg ulcers (VLUs) [34, 35]. Negative Pressure Wound Therapy has been documented to have a role in managing VLUs and can be considered as part of the algorithm of care [36]. Treatment with biological dressings and CAMPs used as an adjunct has also been reported to be associated with higher closure rates than standard of care alone [22, 23, 24, 25, 37, 38].

LPM is a unique shelf‐stable CAMP that is processed by minimal manipulation of the placenta. The lyopreservation process retains the extracellular matrix, growth factors, and native cells of the human amnion, thus avoiding the negative effect associated with techniques such as terminal sterilization, radiation, or dehydration [38, 39, 40]. Previously published data suggests that LPM application may be associated with a decrease in inflammation and oxidative stress, and an increase in angiogenesis with decreased TNF‐α secretion and upregulated VEGF expression [12, 41, 42], which can all manifest as a better clinical outcome when incorporated in the VLU treatment algorithm. In this study, we report that as SoC becomes less effective with increasing VLU wound size, LPM used as an adjunct, results in faster closure and improved quality of life. This is consequential because, wound size and ulcer duration are known negative prognostic factors for healing [27, 28, 43, 44, 45]. Likelihood of VLU closure reduces by 10% with each 1 cm^2^ increase in lesion size [46] and by 3% for each 1‐month increase in the ulcer duration at baseline [46]. While wounds larger than 10 cm^2^ with more than 12 months of duration has a 78% risk of staying open [47], VLUs less than 10 cm^2^ in size and less than 12 months in duration, have a 50%–70% healing rate [47, 48] with standard of care alone. However, as we notice in this study, even in this population, the efficacy of SoC decreases rapidly with an increase in the initial wound size. More than 50% of VLUs fall in this category [47, 49]. This study is uniquely designed to understand the clinical benefits of advanced therapies such as CAMPs, specifically in this population.

To avoid the risk of bias, the study was multicenter and randomized. The outcome assessment was confirmed by blinded evaluators at the Wound Core Lab at CPC Clinical Research. The data also reports outcomes on the ITT population. The demographics of the patient population recruited in this study also align with the US CMS population. A major strength of this study is the reporting on the impact on Quality of Life (QoL). Along with wound progression, QoL enables a more accurate representation of the effect of a specific intervention [50, 51]. QoL, specifically physical components such as pain and physical function, diminishes in patients with VLUs [52, 53, 54, 55]. In comparison to other CAMPs, only one study has reported an impact on QoL [56]. The findings with the use of LPM as reported in this manuscript, thus provide the physicians with a commercially available product that may be associated with improving quality of life for the patients, and ultimately a reduction in health care costs [57].

An important finding from this study demonstrated that there were no differences in complete wound closure in wounds lesser than an initial size of 3 cm^2^, although there was a statistical improvement in wound progression and rate of closure. It is also worth noting that, a number of trials in the last 10 years have failed to report a statistically significant difference in complete wound closure between treatment group and SoC [58], although there is a significant difference in wound size reduction indicating clinical benefits for treating VLUs. This highlights the importance of patient selection and alternate endpoints in the design of VLU trials [59, 60].

### 4.1 Limitations

The main limitation of this study was that blinding of patients and investigators was not possible because of the treatment regimens used in both groups. An additional limitation was sample size. When compared to other reported studies on VLU, our closure rate in the SoC arm appears to be high. On further analysis, we found that wounds between 0 and 3 cm^2^ have > 60% closure rate with SoC alone. Hence, a much higher sample size may be needed to observe statistical difference in full closure, if these smaller wound sizes (which are also, less than a year old) are to be included in the analysis. A recently published study [38], demonstrating statistically improved closure rates, reported similar enrollment criteria and excluded wounds less than 2 cm^2^. A stratification of the data during randomization by taking the initial wound size into consideration and a greater statistical power of the study along with a “center‐effect” in the calculations, considering that this is a multicenter study, would have been a desirable approach. It is worth noting that the study reached statistical significance for wounds ≥ 3 cm^2^ where closure rates for the control group (SoC alone) was around 25%, which is what the assumption was during power analysis. Additionally, we did adjust for loss‐to‐follow up, although it was more than anticipated. Loss‐to‐follow up was 8% and we mitigated it by using the ITT protocol.

## 5 Conclusions

This multicenter, randomized, controlled study provides Level 1 evidence that, LPM as an adjunct to standard of care, significantly closed more venous leg ulcers and faster than standard of care alone and improved the quality of life for patients, suggesting that the use of aseptically processed LPM is a safe and effective treatment option in the healing of chronic venous leg ulcers.

## Author Contributions

**Y. Dhillon:** investigation and methodology. **L. Levine:** investigation and methodology. **G. Tovmassian:** investigation and methodology. **A. Reyzelman:** investigation and methodology. **F. Perez‐Clavijo:** investigation and methodology. **F. Wodie:** investigation and methodology. **S. Cazzell:** investigation and methodology. **A. Grossman:** investigation and methodology. **L. Robinson:** investigation and methodology. **F. Sigal:** investigation and methodology. **R. Kirsner:** investigation and methodology. **M. Vartivarian:** investigation and methodology. **Molly Saunders:** project administration, resources, writing – original draft, writing – review and editing. **Jaideep Banerjee:** formal analysis, writing – original draft, writing – review and editing.

## Ethics Statement

The study was carried out to ensure adherence to GCP; the ethical principles that have their origin in the Declaration of Helsinki; Title 21 of the Code of Federal Regulations §§ 50, 54, and 56, and ICH E6. The study was approved by the IRB of each treating institution.

## Consent

All patients signed an institutional review board (IRB)‐approved informed consent form (ICF) and an authorization for use of protected health information (PHI).

## Conflicts of Interest

J.B. is an employee of Smith & Nephew, which manufactures this allograft. The authors declare no conflicts of interest.

## Transparency Statement

The lead author Jaideep Banerjee affirms that this manuscript is an honest, accurate, and transparent account of the study being reported; that no important aspects of the study have been omitted; and that any discrepancies from the study as planned (and, if relevant, registered) have been explained.

## Data Availability

The data supporting the findings of this study are available within the article and additional information will be available upon request from the corresponding author upon reasonable request.
